# The effect of anesthesia depth on radiofrequency catheter ablation of ventricular tachycardia: a retrospective study

**DOI:** 10.1186/s12871-021-01503-6

**Published:** 2021-11-15

**Authors:** Hongquan Dong, Nana Li, Zhaochu Sun

**Affiliations:** Department of Anesthesiology, The First Affiliated Hospital of Nanjing Medical University/Jiangsu Province Hospital, Nanjing, 210029 China

**Keywords:** Anesthesia depth, Bispectral index, Ventricular tachycardia, Radiofrequency catheter ablation

## Abstract

**Background:**

Radiofrequency catheter ablation (RFCA) as a safe and effective method has been widely used in ventricular tachycardia (VT) patients, and with which anesthesiologists frequently manage their perioperative care. The aim of this study was to investigate the effects of different anesthetic depths on perioperative RFCA and recurrence in patients who with intractable VT and could not tolerate an awake procedure.

**Methods:**

We reviewed electronic medical records of patients with VT who underwent RFCA by general anesthesia from January 2014 to March 2019. According to intraoperative VT induction, they were divided into two groups: non-inducible group (group N) and inducible group (group I). We constructed several multivariable regression models, in which covariates included patient characteristics, comorbidities, protopathy and bispectral index (BIS) value.

**Results:**

One hundred one patients were analyzed. Twenty-nine patients (28.7%) experienced VT no induction, and 26 patients (25.7%) relapsed within 1 year. Compared with group I, the proportion of patients with arrhythmogenic right ventricular cardiomyopathy in group N were higher (*P* < 0.05), and the recurrence rate of VT was significantly higher (51.7% vs 15.3%) (*P* < 0.05). The BIS value in group N was significantly lower (*P* < 0.01), in addition, the BIS < 40 was associated with elevated odds of VT no induction compared with a BIS > 50 (odds ratio, 6.92; 95% confidence interval, 1.47–32.56; *P* = 0.01). VT no induction was an independent predictor of recurrence after RFCA (odds ratio, 5.01; 95% confidence interval, 1.88–13.83; *P* < 0.01).

**Conclusion:**

Lower BIS value during VT induction in RFCA operation was associated with high risk of VT no induction, which affects postoperative outcomes. We proposed that appropriate depth of anesthesia should be maintained during the process of VT induction.

## Background

Ventricular tachycardia (VT) typically arises from structural heart disease, and increases the risk of sudden cardiac arrests in patients with organic heart disease [1, 2]. With the deepening understanding of the pathogenesis of ventricular arrhythmia, radiofrequency catheter ablation (RFCA) has become a first-line treatment for refractory VT [3].

Due to the good sedative and analgesic effects, general anesthesia has been used in RFCA for more complex VT. However, current studies have found that some anesthetics may have the effects of myocardial protection and anti-arrhythmia, which might affect cardiac conduction and interfere with the clinical inducibility of VT [4].

This study aims to compare the effects of different anesthesia depths on the inducibility of VT during RFCA and the postoperative recurrence, which can guide the anesthesiologists to use anesthetics reasonably and help the patients to get through the challenges safely.

## Methods

This retrospective observational study was approved by the Institutional Review Board (IRB) of Jiangsu Province Hospital (JSPH; Jiangsu, China; IRB approval number: 2019-SR-317). Considering the retrospective design of this study, the requirement for informed consent was waived by the IRB.

### Data registry and patient selection

**Fig. 1:**
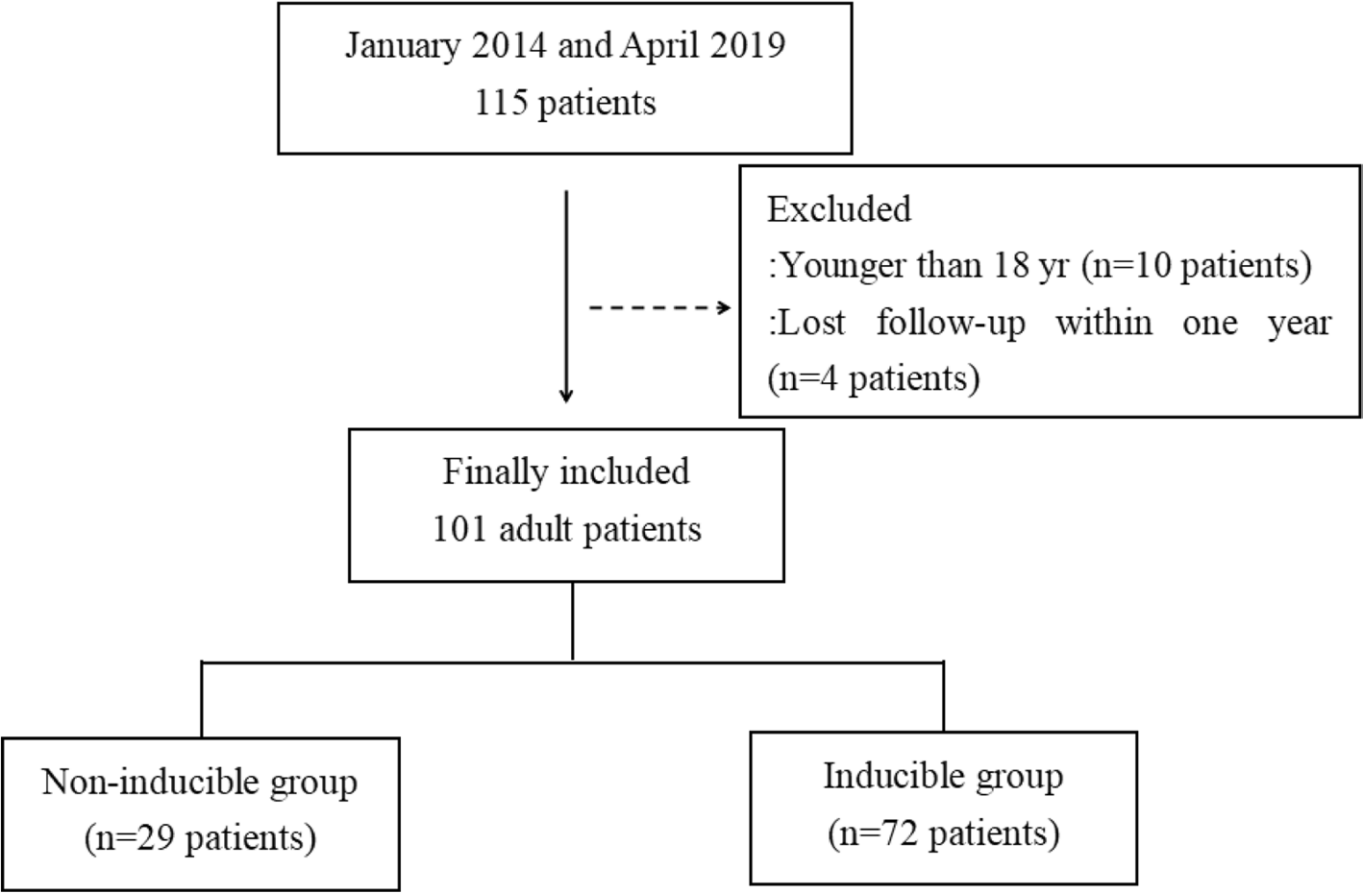
Flowchart summarising the steps used for patient selection

This study utilised data stored and managed in the electronic medical record system of JSPH on the adult patients (18 yr) who were decided to perform RFCA operation under general anesthesia by cardiologist due to VT, between January 2014 and Apirl 2019 (Fig. 1). All the cases for the study period were screened by a group of medical record technicians in the medical informatics team who were not informed of the purpose of this study. Patients who were lost to follow-up within 1 year after surgery, were excluded from the analysis.

To compare the relationship between the success rate of VT inducibility and intraoperative bispectral index (BIS) value, we divided recipients into two groups as follows: non-inducible group (group N) and inducible group (group I). We hypothesized that the BIS values were associated with VT inducibility, which might affect the procedure of operation and recurrence. The primary outcome was assessed the difference of intraoperative BIS between the two groups. We recorded the selected information: gender, age, primary disease, cardiac function, comorbidities; the BIS during VT induction in the two groups, as well as the operative time, ablation time (from the beginning of mapping to the end of ablation), and fluoroscopy time (cumulative exposure time during operation) which recorded in the operation care record sheet by nurse. Patients were followed up regularly for 1 year after the operation, and the recurrence was recorded.

### Monitoring and anesthesia

All operations were performed using standard VT techniques, and intraoperative anesthetic management was performed with continuous monitoring of peripheral capillary oxygen saturation, electrocardiography (ECG), arterial blood pressure. Anesthesia was induced with midazolam 0.05 mg/kg, fentanyl 3 mg/kg, propofol 2 mg/kg, cisatracurium 0.15 mg/kg, and then endotracheal intubation was performed under visual laryngoscope. Mechanical ventilation (Drager, Fabius-Plus, Germany) was used during the operation to keep end-tidal CO2 partial pressure (PetCO2) at 35–45 mmHg. The BIS (Aspect Medical System, Inc., Norwood, Mass, United States) sensor was applied to the patient’s forehead after anesthesia induction, and the BIS was monitored throughout the RFCA procedure. BIS values were recorded per minute from the beginning of programmed VT induction until VT was successfully induced or failed. The median of BIS value during the whole induction process was used as the evaluation index of anesthesia depth. All patients received 1–2% sevoflurane and 2–5 mg/kg·h propofol for anesthesia maintenance until the end of operation, and 0.1 mg/kg·h cisatracurium for muscle relaxation to half an hour before the end. The depth of anesthesia was guided by BIS < 60 to prevent intraoperative awareness.

### Electrophysiological mapping and radiofrequency ablation

#### VT induction

Quadrupole electrode was first used for program stimulation at specific stimulation sites. When VT was not induced, Burst stimulation was performed. In case that VT still wasn’t induced, isoproterenol would be given to increase the heart rate by about 20% and then the above stimulation would be repeated until VT was induced. When all the steps were completed and repeated for three times but it could not be induced, it would be defined as VT no induction.

#### VT mapping

The left ventricular model was constructed under the guidance of 3D electroanatomical mapping system, followed by sequential mapping or pacing mapping. If VT can be persistently and stably induced, sequential mapping were used to find the crucial circuit that will guide ablation during the episode of VT. For non-induced or unstable VTs, pacing mapping were used to reveal scarred regions and potential reentry-circuit channels during sinus or paced rhythm.

#### VT ablation

We used temperature control and non-saline perfusion ablation strategy for VT. After VT disappeared, we observed for 30 min. Then, ventricular program pre-phase stimulation and graded increasing stimulation were performed under conditions of basic state and isoproterenol intravenous injection. When the right ventricular outflow tract ventricular contraction or VT can not be induced, it is regarded as the succeed of immediate ablation.

Postoperative follow-up.

On the second day after ablation, the patients underwent a Dynamic Electrocardiography (DCG). ECG and DCG were reviewed at 3rd, 6th and 12th months postoperative sessions, and the symptoms of VT recurrence were checked by cardiac electrophysiologist to comprehensively determine whether there was VT recurrence.

### Statistical analysis

Statistical analysis was performed with SPSS 20.0 (IBM Software Inc., USA). Categorical variables were presented using numbers with percentages and were analyzed with chi-square test or Fisher’s exact test, whereas the continuous variables were expressed as mean ± standard deviation, and were compared with the Student’s t-test for unpaired samples when a normal deviation was assumed. Univariate and stepwise multivariate logistic regression analysis were performed to determine the risk factors of VT no induction and VT recurrence. All clinically sensible covariates were included in the model. For all analysis, a *P* value of < 0.05 was considered statistically significant.

## Results

We identified a total of 101 patients meeting the inclusion criteria during the study period. The mean age of all patients was 48.7 ± 16.2 years, and the mean LVEF was 53.6 ± 11.7% in the baseline echocardiogram. Twenty two (21.8%) patients had a history of catheter ablation, and 26 (25.7%) experienced the recurrence within 1 year. Patient demographic characteristics are shown in Table 1. There were no differences with baseline characteristics before operation between the two groups. However, the protopathy, BIS values and VT recurrence were different, with more ARVC patients (*P* = 0.03), lower BIS values (*P* < 0.01) and higher recurrence rate of VT (*P* < 0.01) in group N (Table 1).

**Table 1:** Baseline characteristics of 101 patients with a diagnosis of VT and who received RFCA under general anesthesia. Values are number (proportion) or mean (SD)

	Group N(*n* = 29)	Group I(*n* = 72)	*P* Value
Age	45.9 (14.1)	49.9 (16.9)	0.27
Gender (M/F)	27/2	64/8	0.79
BMI (Kg/m^2^)	25.2 (2.6)	24.5 (2.9)	0.24
LVEF	56.3 (11.3)	52.5 (11.7)	0.14
Comorbidity			
Hypertension	6 (20.7%)	20 (27.8%)	0.46
Diabetes mellitus	3 (10.3%)	8 (11.1%)	0.91
Coronary artery disease	2 (6.9%)	13 (18.1%)	0.22
Protopathy			
Dilated cardiomyopathy	7 (24.1%)	24 (33.3%)	0.37
ARVC	17 (58.6%)	25 (34.7%)	0.03*
ICD implantation	12 (41.4%)	41 (56.9%)	0.16
History of VT ablation	4 (13.8%)	18 (25%)	0.29
BIS	46.1 (7.5)	51.2 (5.9)	< 0.01*
Recurrence within 1 year	15 (51.7%)	11 (15.3%)	< 0.01*

*P < 0 .05

The radiation time demonstrated no significant difference between the two groups; however, the difference with operative time and ablation time in group N remained significant longer than those in group I (*P* < 0.05) (Table 2).

**Table 2:** Outcome data of surgical indicators between group A and C, mean and standard deviation are displayed

	Group N(n = 29)	Group I(*n* = 72)	*P* Value
Operative time (min	241.6 (49.5)	219.2 (42.4)	0.02*
Radiation time (min)	18.2 (8.1)	19.9 (6.4)	0.26
Ablation time (min)	156.0 (28.7)	141.4 (25.8)	0.01*

*P < 0 .05

Multivariate logistic regression analysis identified that ARVC (OR, 3.17; 95% CI, 1.23–8.15; *P* = 0.02) and BIS value < 40 (OR, 6.92; 95% CI, 1.47–32.56; *P* = 0.01) were associated with VT non-induction (Table 3). In addition, VT no induction was an independent risk factor (OR, 5.01; 95% CI, 1.88–13.83; *P* < 0.01) for the VT recurrence within 1 year (Table 4).

**Table 3:** Univariate and multivariate logistic regression analysis about VT induction during RFCA

	Univariable		Multivariable	
Variables	OR (95% CI)	*P* Value	OR (95% CI)	*P* Value
Age	1.02 (0.99–1.04)	0.27	–	–
Gender (M)	1.93 (0.39–9.53)	0.42	–	–
LVEF	0.97 (0.93–1.01)	0.14	–	–
Protopathy				
Dilated cardiomyopathy	1.57 (0.59–4.19)	0.37	–	–
ARVC	2.67 (1.10–6.45)	0.03*^a^	3.17 (1.23–8.15)	0.02*
History of VT ablation	2.08 (0.64–6.80)	0.22	–	–
BIS				
> 50	1	–	1	–
40–50	1.38 (0.53–3.62)	0.51^a^	1.74 (0.63–4.80)	0.29
< 40	5.33 (1.23–23.01)	0.03*^a^	6.92 (1.47–32.56)	0.01*
Recurrence within 1 year	5.94 (2.25–15.69)	< 0.01*	5.01 (1.88, 13.83)	< 0.01*

*P < 0 .05

^a^ Analyzed using multivariate analysis

**Table 4:** Univariate and multivariate logistic regression analysis about the recurrence of VT after RFCA

	Univariable		Multivariable	
Variables	OR (95% CI)	*P* Value	OR(95% CI)	*P* Value
Age	0.99 (0.97, 1.02)	0.69	–	–
Gender (M)	3.57 (0.43, 29.67)	0.24	–	–
LVEF	0.99 (0.96, 1.03)	0.71	–	–
Protopathy				
Dilated cardiomyopathy	0.66 (0.26, 1.69)	0.38	–	–
ARVC	1.43 (0.58, 3.54)	0.44	–	–
ICD implantation	0.96 (0.39, 2.35)	0.93	–	–
History of VT ablation	2.56 (0.69, 9.53)	0.16	–	–
BIS				
> 50	1		1	
40–50	1.30 (0.48, 3.57)	0.61^a^	1.19 (0.41, 3.45)	0.75
< 40	6.19 (1.41, 27.24)	0.02*^a^	4.01 (0.81, 19.84)	0.09
Non-induction	5.94 (2.25, 15.69)	< 0.01*^a^	5.01 (1.88, 13.83)	< 0.01*

*P < 0 .05

^a^ Analyzed using multivariate analysis

## Discussion

RFCA is widely used to manage VT associated with structural heart disease when implantable cardioverter defibrillator (ICDs) or antiarrhythmic drugs have failed, and it is usually the sole treatment for idiopathic VT [5]. However, the effect is still unsatisfactory. Some studies have showed that one-year success rate after catheter ablation is 70% [6], and the 5-year recurrence rate is still as high as 46% [7]. In this retrospective study, 101 patients who with intractable VT and operated under general anesthesia were included. Among them, 29 patients (28.7%) experienced VT no induction during operation, and 26 patients (25.7%) relapsed within 1 year. This study also demonstrated that the lower BIS value < 40 was one risk factor for VT no induction, what’s more, the recurrence rate of VT was significantly higher in group N than group I (51.7% vs 15.3%), which shows VT no induction was an independent predictor of VT recurrence within 1 year.

Approaches to sedation in the electrophysiology laboratory range from MAC to general anesthesia (GA). However, electrophysiologists prefer patients awake in the course of arrhythmia induction. To maintain patients comfort and serenity, a low dose sedative agent can be administered. European Heart Rhythm Association (EHRA) suggests avoiding GA, deeper sedations to prevent unsuccessful VT stimulation [8]. Nonetheless, unstable hemodynamics, patient comorbidities, and long lasting transactions VT ablations, GA must be the preferred option to maintain patients comfort during process [9]. All methods of anesthesia have advantages and disadvantages, GA can ensure the stability of the ablation process, while MAC may reduce the impact of drugs on the heart. One retrospective study also showed that, there was no difference in VT inducibility, complications, or abolition of clinical VT in the GA group []. Therefore, we suspect that the depth of anesthesia is a factor affecting the induction of VT rather than the method. The results of this study showed a 6.92 fold pooled risk of VT no induction when BIS value < 40 is compared to BIS value > 50.

The transition from deep sedation to general anesthesia is rather vague, Bispectral Index (BIS), a parameter derived from the electroencephalogram (EEG) parameter that was developed specifically to quantitative measure patient response during the administration of anesthetics and sedatives. It has become one of the most widely used EEG monitoring indexes in clinical. In this study, BIS values were used to distinguish the depth of anesthesia, so as to explore the influence of different anesthesia depths on the induction and prognosis of VT. Clinically, we generally consider BIS value < 40 as deep anesthesia, while BIS value > 60 as light anesthesia. In order to avoid intraoperative awareness, we always keep the BIS value at < 60 in all patients, while for BIS value > 60, whether VT is more likely to induce remains unknown. This evaluation index has not been studied in this field and may provide better guidance for anesthesiologists and electrophysiologists for RFCA. However, the BIS values are likely to be affected by other factors, resulting in inevitable inaccuracy and lag, so cerebral state index (CSI) might be a better choice [11].

The inducibility of arrhythmia is important, much ablation for VT targets symptomatic focal PVCs rather than sustained VT. Furthermore, entrainment mapping and searching for the earliest PP are still the most widely used techniques [12, 13], which can only be used when VT induced stability. Therefore, this study showed that, to control the BIS value > 40 or higher, ensure the induction of arrhythmia and hemodynamic stability plays a very important role in reduce the recurrence of VT during ablation treatment.

It cannot be denied that, many anesthetics may inhibit induction of some arrhythmias and removes the ability to monitor neurologic status during sustained VT, and some types of VT (such as outflow tract VT caused by ARVC) are extremely sensitive to sedation [3]. This study showed that the incidence of VT no induction in patients with ARVC was significantly higher..

Propofol is the most preferred agent provides both sedation and GA in ablation procedure. Studies have demonstrated that propofol has a protective effect on terminate atrial fibrillation and VT storm [14, 15]. What’s more, propofol can also shorten the Q-T interval of long Q-T syndrome; therefore, it may have the potential to prevent episodes of VT which are caused by Q-T interval dispersion [16].

Compared to propofol, volatile anesthetics such as sevoflurane has been showed that has some prolongation effects on QT interval, and reduce the possibility of inducing ventricular arrhythmia in vitro by prolonging the duration of the action potential, but its clinical significance is not clear [17].

Our study has several limitations. First, this study is a retrospective study, in which only BIS values were used as the evaluation index for anesthesia depth. It is difficult to track the real-time blood concentration of various anesthetics, and it is impossible to determine which drug is the main factor that causes non-inducible of VT. Our team will conduct a prospective group analysis of the influence factors in the follow-up prospective research. Second, the number of cases in this study is small and it is a single-center study, which may lead to the bias of results and a large 95% CI.

## Conclusions

In conclusion, we have reported that patients with VT who experience RFCA under general anesthesia, with the increase of anesthesia depth, may increase the risk of VT no induction, which in turn affects postoperative outcomes. Anesthesiologists need to have a deeper understanding of the effects of anesthesia methods and drugs on RFCA, so as to choose the ideal anesthesia management strategy and anesthesia depth to ensure the patient safety while improving the success rate of surgery.

## Data Availability

The datasets used and/or analyzed during this study are available from the corresponding author on reasonable request.

## Abbreviations

RFCA: Radiofrequency catheter ablation
VT: Ventricular tachycardia
BIS: Bispectral index
BMI: Body mass index
LVEF: Left ventricular ejection fraction
ARVC: Arrhythmogenic right ventricular cardiomyopathy
ICD: Implantable cardioverter-defibrillator
GA: General anesthesia
CSI: Cerebral state index

## References

[1] Priori SG, Blomström-Lundqvist C, Mazzanti A (2015). 2015 ESC guidelines for the management of patients with ven- tricular arrhythmias and the prevention of sudden cardiac death. Eur Heart J.

[2] Al-Khatib SM, Stevenson WG, Ackerman MJ (2018). 2017 AHA/ACC/HRS guideline for management of patients with ventricular arrhythmias and the prevention of sudden cardiac death: a report of the American College of Cardiology/American Heart Association task force on clinical practice guidelines and the Heart Rhythm Society. Heart Rhythm.

[3] Deng Y, Naeini PS, Razavi M (2016). Anesthetic Management in Radiofrequency Catheter Ablation of ventricular tachycardia. Tex Heart Inst J.

[4] Haqqani HM, Roberts-Thomson KC (2012). Radiofrequency catheter ablation for ventricular tachycardia. Heart Lung Circulation.

[5] Pedersen CT, Kay GN, Kalman J (2014). EHRA/HRS/APHRS expert consensus on ventricular arrhythmias. Heart Rhythm.

[6] Tung R, Vaseghi M, Frankel DS (2015). Freedom from recurrent ventricular tachycardia after catheter ablation is associated with improved survival in patients with structural heart disease:an international VT ablation center collaborative group study. Heart Rhythm.

[7] Donateo P, Bottoni N, Oddone D (2016). Long-term results after single and multiple procedures of ablation of ventricular tachycardia. J Cardiovasc Electrophysiol.

[8] Aliot EM, Stevenson WG, Almendral-Garrote JM (2009). EHRA/HRS expert consensus on catheter ablation of ventricular arrhythmias: developed in a partnership with the European heart rhythm association (EHRA), a registered branch of the European Society of Cardiology (ESC), and the Heart Rhythm Society (HRS); in collaboration with the American College of Cardiology (ACC) and the American Heart Association (AHA). Heart Rhythm.

[9] Mittnacht AJ, Dukkipati S, Mahajan A (2015). Ventricular tachycardia ablation: a comprehensive review for anesthesiologists. Anesth Analg.

[10] Killu AM, Sugrue A, Munger TM, et al. Impact of sedation vs. general anaesthesia on percutaneous epicardial access safety and procedural outcomes. Europace. 2018;20:329–36. 10.1093/europace/euw313 28339558

[11] Jensen EW, Litvan H, Revuelta M (2006). Cerebral state index during propofol anesthesia: a comparison with the bispectral index and the A-line ARX index. Anesthesiology.

[12] Sung R, Scheinman M (2016). Spectrum of fascicular arrhythmias. Cardiac Electrophysiol Clin.

[13] Liu Y, Fang Z, Yang B (2015). Catheter ablation of fascicular ventricular tachycardia: long-term clinical outcomes and mechanisms of recurrence. Circ Arrhythm Electrophysiol.

[14] Miro O, de la Red G, Fontanals J (2000). Cessation of paroxysmal atrial fibrillation during acute intravenous propofol administration. Anesthesiology.

[15] Mulpuru SK, Patel DV, Wilbur SL (2008). Electrical storm and termination with propofol therapy: a case report. Int J Cardiol.

[16] Kleinsasser A, Loeckinger A, Lindner KH (2001). Reversing sevoflurane-associated Q-Tc prolongation by changing to propofol. Anaesthesia.

[17] Terao Y, Higashijima U, Toyoda T (2016). The effects of intravenous anesthetics on QT interval during anesthetic induction with sevoflurane. J Anesth.

